# Extensive transmission of isoniazid resistant *M. tuberculosis* and its association with increased multidrug-resistant TB in two rural counties of eastern China: A molecular epidemiological study

**DOI:** 10.1186/1471-2334-10-43

**Published:** 2010-02-28

**Authors:** Yi Hu, Sven Hoffner, Weili Jiang, Weibing Wang, Biao Xu

**Affiliations:** 1 Department of Epidemiology, School of Public Health, Fudan University, Shanghai, China; 2 Department of Bacteriology, Swedish Institute for Infectious Disease Control, Stockholm, Sweden

## Abstract

**Background:**

The aim of this study was to investigate the molecular characteristics of isoniazid resistant *Mycobacterium tuberculosis *(MTB), as well as its contribution to the dissemination of multi-drug resistant TB (MDR-TB) in rural areas of eastern China.

**Methods:**

A population-based epidemiological study was conducted in two rural counties of eastern China from 2004 to 2005. In total, 131 isoniazid resistant MTB isolates were molecularly characterized by DNA sequencing and genotyped by IS*6110 *restriction fragment length polymorphism (RFLP) and spoligotyping.

**Results:**

The *katG*315Thr mutation was observed in 74 of 131 isoniazid resistant isolates and more likely to be MDR-TB (48.6%) and have mutations in *rpoB *gene (47.3%). Spoligotyping identified 80.2% of isoniazid resistant MTB isolates as belonging to the Beijing family. Cluster analysis by genotyping based on IS*6110 *RFLP, showed that 48.1% isoniazid resistant isolates were grouped into 26 clusters and *katG*315Thr mutants had a significantly higher clustering proportion compared to those with *katG *wild type (73%.vs.18%; OR, 12.70; 95%CI, 6.357-14.80). Thirty-one of the 53 MDR-TB isolates were observed in 19 clusters. Of these clusters, isoniazid resistance in MDR-TB isolates was all due to the *katG*315Thr mutation; 18 clusters also contained mono-isoniazid resistant and other isoniazid resistant isolates.

**Conclusions:**

These results highlighted that isoniazid resistant MTB especially with *katG*315Thr is likely to be clustered in a community, develop extra resistance to rifampicin and become MDR-TB in Chinese rural settings.

## Background

Isoniazid (INH) is one of the most effective and specific agents for the treatment of the disease caused by *Mycobacterium tuberculosis *(MTB). It is a cornerstone of the modern short-course chemotherapy for tuberculosis, and also widely used to treat the latent MTB infection (LTBI) to prevent the active disease and the subsequent TB transmission.

However, recent increases both in INH-resistant and multidrug-resistant (MDR) tuberculosis have been jeopardizing the efforts of global TB control through the implementation of the Directly Observed Treatment, Short course (DOTS) [1,2]. In China, DOTS program has been adopted since early 1990s, but the increased prevalence of drug resistant TB has become a significant challenge for TB control in last ten years. The prevalence of MDR-TB and INH resistant TB was estimated as 8.3% and 41.2% respectively among all cases in China, which were both high compared to the global estimates of 4.8% and 13.3% [3].

The development of INH resistance is a common first step in the evolution to MDR [4]. Thus, there has been considerable interest in both identifying the molecular basis of INH resistance and understanding the transmission pattern of the INH resistant MTB strain.

Resistance to INH is mediated by mutations in different genes in MTB, with *katG *[5] and *inhA *[6] being the most common. The mutation in the *katG *gene is a major mechanism of INH resistance in MTB [7-9]. The most common mutation is the Ser315Thr substitution in the *katG *gene, which is present in approximately 50-90% of all INH-resistant isolates and is associated with relatively high-level resistance to INH [9]. The mutation in *inhA *or its promoter region can cause 21-24% of INH resistance, with the promoter mutation (mainly in the *inhA*-15 position) being more common than the mutation in the structural gene [8]. A study of INH resistance in MTB has reported that a certain INH resistant strain with the *katG*315Thr mutation had a strong association with the development of MDR, and the successful transmission of MDR MTB as well [10].

Based on the above knowledge and findings, we hypothesized that specific INH resistant MTB strains could perpetuate the epidemic of MDR-TB in high TB burden countries like China.

Here we presented an in-depth study to determine the molecular basis of INH-resistant MTB and the transmission pattern of INH resistant MTB circulating in two rural counties of eastern China. We applied direct DNA sequencing on hotspots of *katG *and *inhA *genes/promoters as well as the IS*6110 *restriction fragment length polymorphism (RFLP) genotyping technique to analyze the level of clustering or recent transmission of INH-resistant MTB. Combined with epidemiological evidence, the molecular information obtained from this study could give us a better understanding of the possible mechanism behind the high prevalence of MDR-TB in rural China.

## Methods

### Study population

The epidemiological methods and study population of this study were described previously [11]. It is a population-based cross-sectional study carried out in two rural counties in eastern China, i.e., Deqing County in Zhejiang Province and Guanyun County in Jiangsu Province. All TB patients notified in the County TB dispensaries within one year were enrolled in the study (Deqing, from April 1, 2004 to March 31, 2005; Guanyun, from June 1, 2004 to May 31, 2005).

### Drug susceptibility test

All MTB isolates were tested for drug susceptibility using the proportion method on egg-based LJ medium [12]. The critical drug concentration were 0.2 μg/ml for isoniazid (INH), 40 μg/ml for rifampicin (RIF), 4 μg/ml for streptomycin (STR) and 2 μg/ml for ethambutol (EMB). Resistance was defined as the growth of more than 1% colonies compared to the drug free control. MDR was defined as drug resistance to at least INH and RIF. Poly drug-resistance was defined as resistance to more than one of the tested anti-TB drugs but not to both INH and RIF simultaneously.

### DNA sequencing

All isolates available were investigated for the presence of the hotspot mutations related to drug resistance by direct DNA sequencing. The hotspot genes included *rpoB*, *katG *genes and the *inhA *promoter region, corresponding to the drug resistance to RIF and INH respectively. The Primers pairs were CCCATGGCCGCGGCGGTCGACATT and CGCCGTCCTTGGCGGTGTATTGCC for *katG *gene (GeneBank: X68081), and CCTCGCTGCCCAGAAAGGGA and ATCCCCCGGTTTCCTCCGGT for *inhA *promoter region (GeneBank: U41388), and GGGAGCGGATGACCACCCA and GCGGTACGGCGTTTCGATGAAC for *rpoB *gene (GeneBank: L27989). Mutations in these genes were determined by amplification of the corresponding hotspot DNA region by PCR followed by direct DNA sequencing with ABI 3770 DNA sequencer (Applied Biosystems, Inc., Foster City, CA, USA). The new alleles were confirmed by further PCR and re-sequencing from the original DNA. Sequencing data was independently analyzed by two biologists for quality control purposes.

### IS*6110*-RFLP genotyping

IS*6110 *RFLP was done on the INH resistant isolates according to the standard protocol of van Embden et al, with data analyzed by the Gel Compar software (version 4.6, Applied Maths, Belgium) [13]. A cluster is defined as two patient MTB isolates harboring the identical IS*6110 *RFLP pattern. Unique strains denote unparallelness of the IS*6110 *RFLP profiles in the study collection. Typically, clustered strains indicate recent transmission while unique strains indicate reactive disease from a remote infection.

### Spoligotyping

Spoligotyping was carried out by using the commercial kit from Isogen Bioscience BV (Maarssen, The Netherlands) [10]. The INH resistant strain clades were determined by comparison of the spoligotyping pattern with the SpolDB3 database [14] in the SpotClust program (available in http://cgi2.cs.rpi.edu/~bennek/SPOTCLUST.html) and assigned with the corresponding spoligotype international type (SIT). The Beijing family MTB was defined as the strain that hybridized only to the last nine spacer oligonucleotides (spacers 35 to 43).

### Statistics analysis

SPSS software (SPSS Inc., Chicago, IL, USA) was utilized for the statistics analysis. Clinical and bacteriological characteristics were compared between *katG*315Thr alleles and other INH resistant isolates using the Mantel-Haenszel chi-square test. Binary logistic regression model was used for univariate and multivariate analysis to qualify and quantify the difference in clustering proportion between groups of subjects with different socio-demographic and clinical characteristics. The adjusted Odds Ratio (OR) and 95% confidence interval (CI) were calculated by adjusting for the possible confounders (age, county and sex).

The study was approved by the Institutional Review Board of Fudan School of Public Health. Written informed consent was obtained from all the participants.

## Results

In total, drug resistance profile and baseline information were successfully obtained from 399 pulmonary TB patients, 182 in Deqing and 217 in Guanyun during the study period. Of the 399 MTB isolates from these patients, 251 (62.9%) were resistant to at least one of 1^st ^line anti-TB drugs. Resistance to INH was the most common form of drug resistance (140/251) with a majority of cases mono-resistant to INH (55/140). Resistance to RIF was observed in 65 isolates, only 2 of which were mono-resistant. A total of 58 isolates were resistant to INH and RIF simultaneously. The details of the resistance have been reported previously [11].

DNA samples were successfully extracted from 131 of the 140 INH resistant isolates. Of the 131 isolates, 80 were from patients who were previously diagnosed with TB and 51 were from the patients newly diagnosed. Regarding the drug resistance profile, apart from the 55 INH mono-resistant isolates, 53 were also resistant to RIF and referred to as MDR-TB; 23 were poly-resistant (INH + STR and/or EMB) (Table 1).

**Table 1:** Drug susceptibility profile of INH resistant MTB isolates from the study sites

Drug resistance profile	Total *n *= 131	No. of isolates from patients with:	
		
		newly diagnosed TB *n *= 80	previously diagnosed TB *n *= 51
**H**	**55**	**30**	**25**
**MDR-TB**	**53**	**33**	**20**
HR	32	21	11
HRS	12	6	6
HRE	5	3	2
HRSE	4	3	1
**PDR-TB**	**23**	**17**	**6**
HS	13	9	4
HE	2	1	1
HSE	8	7	1

NOTE: H, isoniazid; R, rifampicin; S, streptomycin; E, ethambutol;

MDR-TB, multidrug-resistant tuberculosis; PDR-TB, polydrug-resistant tuberculosis.

DNA sequencing demonstrated that 107 of 131 INH resistant isolates had mutations either in the *katG *gene or in the *inhA *promoter (Table 2): 61.8% (81/131) of the isolates had a mutation in the position 315 of *katG*, of which 56.5% (74/131) INH resistant isolates contained the *katG *Ser315Thr nucleotide substitution. *katG*315Arg and *katG*315Asn mutations were detected in 3 and 4 isolates respectively. In addition, 28 of 131 INH resistant isolates (21.4%) presented the mutation in the *inhA *promoter region, all of which possessed the *inhA*-15 C→T nucleotide substitution. Two INH resistant isolates shared the mutation in *katG *and *inhA *gene simultaneously: one with *katG *315Thr and *inhA*-15T mutations and the other with *katG*315Arg and *inhA*-15T mutations. No nucleotide substitutions were identified in 220 INH sensitive isolates either at the *katG*315 position or the *inhA*-15 position. The frequency of the *katG*315Thr mutation was significantly higher in MDR-TB compared to the other drug resistant form of MTB isolates (67.9%.vs.48.7%; χ^2^, 4.736; *p*, 0.030), while *inhA*-15T mutations did not differ significantly between these two major drug susceptibility groups (20.8%.vs.21.8%; χ^2^, 0.020; *p*, 0.887).

**Table 2:** Genetic mutations and their frequencies related to INH resistance in MTB isolates from the study sites

Drug resistance	Total	*katG*315Ser → *			*inhA *-15C→ *	*katG *+	wt
					
Profile		Arg	Asn	Thr	T	*inhA*^§^	
H	55	2	1	28	12	0	12
**MDR-TB**							
HR	32	0	1	19	6	1	5
HRS	12	0	0	9	2	1	0
HRE	5	0	0	5	0	0	0
HRSE	4	0	1	2	1	0	0
**PDR-TB**							
HS	13	0	0	5	3	0	5
HE	2	0	0	0	1	0	1
HSE	8	0	1	5	1	0	1

NOTE: H, isoniazid; R, rifampicin; S, streptomycin; E, ethambutol.

MDR-TB, multidrug-resistant tuberculosis; PDR-TB, polydrug-resistant tuberculosis.

*: No. of isolates with mutations in single gene.

§: No. of isolates with mutations in multiple genes. Two INH resistant isolates shared the mutation in *katG *and *inhA *gene simultaneously: one with *katG*315Thr and *inhA*-15T mutations and the other with *katG*315Arg and *inhA*-15T mutation.

The resistance to RIF was due to mutations in the *rpoB *gene in 49 of 53 MDR-TB isolates. A single-nucleotide substitution in position 516, 526 and 531 accounted for 7.5%, 30.2% and 58.5% respectively of MDR-TB. The mutations in *rpoB *gene included 516Tyr (3/53), 516Val (2/53), 526Arg (4/53), 526Tyr (11/53) and 531Leu (31/53). Double-spot mutations were presented in two MDR isolates, one with 516Tyr/531Leu mutations and the other with 516Val/531Leu mutations. No *rpoB *mutations were observed in isolates susceptible to RIF.

MTB clades designations were available for 122 of 131 isolates, with the following distribution of the genotypic lineages (Table 3): Beijing family (105/131 or 80.1%), Family 33 (8/131 or 6.1%), T lineage (7/131 or 5.3%), Haarlem (1/131 or 0.8%) and LAM (1/131 or 0.8%). Of the remaining 9 isolates, 6 had the spoligotyping pattern similar to T lineage, 1 similar to Family 33, 1 similar to Haarlem and 1 similar to LAM.

**Table 3:** Spoligotyping pattern and frequency of the MTB isolates included

Octal designation	Clade*	Probability^§^	SIT	No. of isolates
0000 0000 0003 771	Beijing	0.99	1	105
5777 7777 7760 771	T1	0.99	334	2
7777 7777 7760 031	T1	0.99	239	1
7777 7777 7760 771	T1	0.99	53	4
7777 7736 7730 771	T1	0.79	new	1
7777 2737 7730 771	T1	0.79	new	1
7777 7777 7730 771	T1	0.79	2597	1
7577 3737 7730 771	T1	0.79	new	1
7736 3737 7730 771	T1	0.79	new	1
7736 7777 7730 771	T1	0.79	new	1
7777 7777 7146 741	Family33	0.99	new	1
5777 7763 3566 731	Family33	0.99	new	1
7767 6767 1146 771	family33	0.99	new	1
7777 7377 7731 771	family33	0.99	new	2
7777 7777 1146 771	family33	0.99	new	1
7777 3717 7733 571	Family33	0.99	new	1
7777 3771 7731 761	Family33	0.99	new	1
7777 7777 7630 771	Family33	0.70	new	1
7777 7777 6000 371	Haarlem1	0.98	1498	1
5777 7777 7700 771	Haarlem3	0.77	new	1
7777 7760 3560 731	LAM9	0.99	new	1
6777 7760 7560 771	LAM1	0.51	1755	1

NOTE: SIT, spoligotype international type.

*: Spotclust program-assigned clade.

§: Probability that the spoligotyping pattern belongs to the clade.

**Figure 1:**
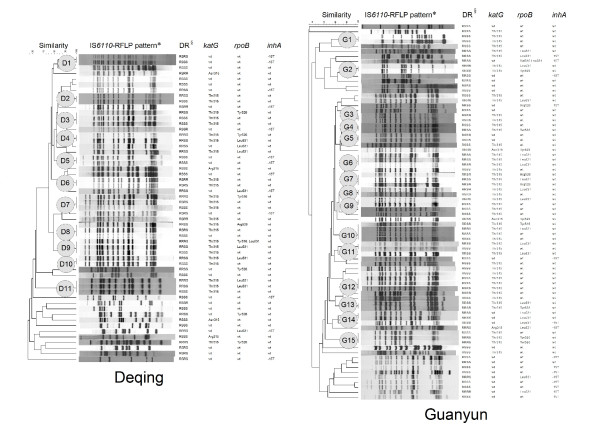
**The phenotypes and genotypes of INH resistant MTB isolates with cluster and unique pattern respectively in Deqing and Guanyun**. NOTE. DR, 1^st ^line Drug resistance profile. *: The circle contained the code for the cluster. §: Sequence of drugs was isoniazid, rifampicin, streptomycin, ethambutol; R, resistant; S, susceptible.

In the clustering analysis of INH resistant isolates specific to counties (Figure 1), IS*6110 *RFLP identified all together 94 IS*6110 *RFLP patterns, including 11 cluster patterns (27 isolates), 28 unique patterns/isolates in Deqing, 15 cluster patterns (36 isolates) and 40 unique patterns/isolates in Guanyun. The genotype and phenotype patterns of drug resistant TB were further investigated among the clustered isolates. Ten clusters (25 isolates) from Deqing and 12 clusters (32 isolates) from Guanyun contained isolates with different phenotype and/or genotype of drug resistance. In these clusters, INH resistant isolates with the *katG*315Thr mutation had the highest occurrence (51/57 or 89.5%). Additionally, 31 of 53 MDR-TB isolates (58.5%) were observed in 19 clusters, with all carrying the *katG*315Thr mutation as well as the mutations either in position 516, 526 or 531 of the *rpoB *gene. In these clusters containing MDR-TB isolates, 18 clusters had INH-mono resistant and/or polydrug-resistant TB isolates simultaneously.

Binary logistic regression was applied to analyze the "clustering" of INH resistant isolates in association with patients' demographics, clinic profiles and the bacteriologic features (Table 4). Smear-positive TB patients had a significantly higher proportion of clustered INH resistant MTB strain than smear-negative patients (54.9%.vs.36.7%; adjusted OR, 2.286; *p*, 0.032; 95%CI,1.075-4.863), as well as the previously diagnosed patients when compared to newly diagnosed patients (60.8%.vs.40.0%; adjusted OR, 2.159; *p*, 0.040; 95%CI,1.037-4.495). A significant higher proportion of MDR-TB isolates was clustered compared to INH mono resistant isolates (58.5%.vs.38.2%; adjusted OR, 2.384; *p*, 0.030; 95%CI, 1.089-5.220). Compared with the wild type (wt) isolates, the isolates with the *katG*315Thr mutation were more likely to be clustered (73.0%.vs.18.0%; adjusted OR, 12.70; *p*, 0.001; 95%CI, 6.357-14.80). The Beijing family isolates was mainly observed in clusters (56.2%.vs.15.4%; adjusted OR, 5.289; *p*, 0.001; 95%CI, 1.832-15.26). The INH resistant *inhA*-15T mutant isolates were more likely to be "unique" compared to those *inhA*-15 wild type isolates (14.3%.vs.57.3%; adjusted OR, 0.120; *p*, 0.001; 95CI%, 0.038-0.375).

**Table 4:** Binary logistic regression analysis on the associations between demographical, clinical and bacteriological characteristics and clustering of INH resistant MTB isolates in the present study

Variables		INH resistant isolates		unadjusted OR	adjusted OR*	*p*	95%CI
						
		No. patients	*n *(%) clustered				
**Social-demographic and clinical characteristics:**							
Age (year)	*18~*	34	12(35.3)	1			
	*30~*	65	34(52.3)	2.011			
	*60~*	32	17(53.1)	2.078			
Sex	*Female*	46	22(47.8)	1			
	*Male*	85	41(48.2)	1.163			
County	*Deqing*	55	27(49.1)	1			
	*Guanyun*	76	36(47.4)	0.933			
BMI	≥*18.5*	94	41(43.6)	1	1		
Index	<*18.5*	37	22(59.5)	1.896	1.833	0.13	0.836-4.019
Treatment	*New*	80	32(40.0)	1	1		
history	*Previously*	51	31(60.8)	2.325	2.159	0.040^§^	1.037-4.495
Sputum	*negative*	49	18(36.7)	1	1		
smear	*positive*	82	45(54.9)	2.095	2.286	0.032^§^	1.075-4.863
Cavity	*No*	111	50(45.0)	1	1		
	*Yes*	20	13(65.0)	2.266	2.423	0.091	0.868-6.761
**Bacteriological features:**							
Drug	*INH mono*	55	21(38.2)	1	1		
resistance	*MDR-TB*	53	31(58.5)	2.281	2.384	0.030^§^	1.089-5.220
	*PDR -TB*	23	11(47.8)	1.484	1.627	0.348	0.588-4.496
*katG*	*wt*	50	9(18.0)	1	1		
	*315Thr*	74	54(73.0)	12.3	12.7	0.001^§^	6.357-14.80
	*others*	7	0(0)	-	-		
*inhA*	*wt*	103	59(57.3)	1	1		
	*-15T*	28	4(14.3)	0.124	0.12	0.001^§^	0.038-0.375
Beijing	*No*	26	4(15.4)	1	1		
genotype	*Yes*	105	59(56.2)	5.183	5.289	0.001^§^	1.832-15.26

NOTE. OR, odds ratio; 95%CI, confidence interval; wt, wild type; MDR-TB, multidrug-resistant tuberculosis; PDR-TB, polydrug-resistant tuberculosis.

*: OR and 95%CI were adjusted by age, sex and county of subjects in binary logistic regression model.

§: *p *< 0.05.

To further investigate the characteristics of *katG*315Thr mutant, the host and bacteriological variable were compared between the INH resistant isolates with the *katG*315Thr mutation group and those with other mutation/wild type group (Table 5). The *katG*315Thr mutant isolates had a significantly higher proportion of MDR-TB (48.6%.vs.29.8%; *p*, 0.03), the relevant mutation in *rpoB *gene (47.3%.vs.24.6%, *p*, 0.008) compared to other mutation/wild type group. And meanwhile, this allele was more likely to be clustered (73.0%.vs.15.8%; *p*, 0.001) and belonged to the Beijing family (87.8%.vs. 70.2%; *p*, 0.012).

**Table 5:** Comparison of host and bacteriological characteristics between isoniazid resistant isolates with katG315Thr mutations and other mutation/wild type

		No. of isolates with:			
				
Variables		*katG*315Thr *n *= 74	Others *n *= 57	χ^2^	*p*
Sputum smear	*Negative*	23(31.1)	26(45.6)	2.905	0.088
	*Positive*	51(68.9)	31(54.4)		
Treatment history	*New*	41(55.4)	39(68.4)	2.294	0.13
	*Previously*	33(44.6)	18(31.6)		
Cavity	*No*	59(79.7)	52(91.2)	3.291	0.07
	*Yes*	15(20.3)	5(8.8)		
Drug resistance profile	*Others*	38(51.4)	40(70.2)	4.736	0.030*
	*MDR-TB*	36(48.6)	17(29.8)		
*rpoB *mutation	*No*	39(52.7)	43(75.4)	7.11	0.008*
	*Yes*	35(47.3)	14 (24.6)		
Clustered	*No*	20(27.0)	48(84.2)	42.18	0.001*
	*Yes*	54(73.0)	9(15.8)		
Beijing family	*No*	9(12.2)	17(29.8)	6.314	0.012*
	*Yes*	65(87.8)	40(70.2)		

*: *p *< 0.05

## Discussion

This study attempted to gain further insight into the molecular basis of the INH resistant MTB circulating in the rural area of eastern China. Transmission of INH resistant MTB and its possible contribution to the epidemic of MDR-TB were also a concern. The majority of INH strains from TB dispensaries in the rural Chinese area, exhibited INH mono-resistance and the double-drug-resistance profile especially including extra resistance to RIF (53/131) and STR (37/131). These resistance profiles corroborated the pattern of acquisition of resistance to extra drug resistance especially for RIF and STR in INH resistant isolates [1].

The prevalence of the *katG*315Thr mutation in MTB strains around the world varies, especially with regard to the prevalence of TB. High TB burden regions [15-17] always observe a higher prevalence of the *katG*315Thr mutation in INH resistant strain compared to low TB burden areas [18-20]. The present study also demonstrated a high prevalence of the *katG *315Thr mutant allele in eastern rural China in around 50% of INH resistant isolates, which is consistent with the other report from China [21]. Furthermore in the current study, a significant correlation was confirmed between the common *katG*315Thr mutation in INH resistant strains and the presence of resistance to other drugs, especially for RIF. These observations might suggest that the INH resistant strain with the *katG*315Thr mutation may be more likely to develop the resistance to other 1^st ^line drugs especially for RIF [22-24] than the strains with other forms of INH-resistance. Meanwhile, the high proportion of INH resistant *katG *315Thr mutant allele bore the mutation in *rpoB *gene, which was related to RIF resistance reported in China [25] and could be the reasons for the high risk of developing MDR in this allele.

The extensive transmission of the INH resistant MTB strain was observed in rural areas of eastern China, with 48.1% of the INH resistant MTB isolates in clusters. Most of INH resistant isolates with *katG *mutations were in clusters compared to wild type INH resistant isolates, suggesting its strong transmissibility, full virulence and survival advantage under the drug pressure in the host. This could be explained by the maintenance of the 30%~40% catalase-peroxidase activity in the INH resistant strain with the *katG *315 Thr mutation [5,6] compared to other mutations related to drug resistance. Consequently, this allele was suspected as the cause of some earlier reported outbreaks [26-28] of INH resistant TB in specific areas and populations. Furthermore, the current study observed that in a cluster containing strains with different drug resistance profile and resistance conferring mutations, the INH resistance was caused by this mutation. This increased the possibility that the INH resistant MTB strain with the *katG *315 mutation in the index person was more likely to experience a series of subsequent mutations, leading to the accumulation of extra drug resistance and the development of MDR- and/or extreme drug resistant(XDR)-TB [29]. The accumulation of drug resistance could be accredited by less efficiencies of standard chemotherapy, the subsequent mutation of drug resistance-related gene and selective survival of drug resistant strain during the recent transmission.

Apart from the role of the *katG*315Thr mutation, the transmissibility of INH resistant strain might be related to endemic characteristics of the Beijing family in rural China. The Beijing genotype is apparently the most prevalent genotype in China, representing of 80.2% INH resistant and 83% MDR-TB isolates in the current study. Our study also found that the MTB strain from the Beijing family was also the main source of recent transmission causing INH resistant TB. The Beijing genotype has been reported to be associated with treatment failure and relapse. Although the reasons for this are not known, it appears that the infections with the Beijing genotype strain may be more persistent, probably leading to longer duration of infectiousness and creating a better opportunity for transmission under the anti-TB drug pressure [30].

Additionally in the current study, INH resistant MTB was more likely to be clustered in smear-positive and previously diagnosed patients. The smear-positive status might extend the transmission period of the pathogen between hosts and allow MTB to attack more people in the surroundings of the index case. Although the previous TB could be cured by direct observed chemotherapy, those formerly treated cases could still be at risk for the TB re-infection from recent transmissions since the risk factors for TB (low socio-economic status, exposure to MTB etc.) are still present. In combination with these conditions, the risk of INH resistant MTB transmission between hosts increases.

The epidemic of MDR-TB can occur as a result of the spontaneous mutations in MTB and selection under suboptimal drug therapy or extensive transmission of the drug resistant MTB, or both combined. In the current study, a significant high proportion of MDR-TB isolates were observed in clusters compared to the other form of INH mono-resistant isolates. Most clusters with MDR-TB isolates also contained isolates with other drug resistance profiles including INH mono-resistance and poly drug-resistant tuberculosis simultaneously. Based on these observations, the epidemic of MDR-TB in rural China could be explained by two possibilities: 1) the epidemic of MDR-TB might result from the recent transmission of MDR-TB strains in an area ridden by INH resistant MTB. 2) Recent transmission of the drug resistant MTB and the selection of MTB under drug pressure might exert the symbiotic interaction in an epidemic of MDR-TB. The combination of INH resistance and maintained virulence might make it possible for some INH resistant strains, especially those with the *katG*315Thr mutation, to acquire extra drug resistance and become MDR-TB. It deserves further investigation to determine which mechanism may play the critical role in the epidemic of MDR-TB, since the implication behind it could be meaningful to evaluate the performance of local TB control as well as to determine the MDR-TB control strategies suitable for rural areas of China and as well as other similar high burden settings.

## Conclusions

INH resistant MTB was transmitted widely in eastern rural areas of China. Also the correlation of prevalence and transmission between INH resistant isolates especially with the *katG*315Thr mutation and MDR-TB was confirmed. Therefore, it is important to recognize the *katG*315Thr mutants among INH-resistant strains, which could be seen as a risk factor for subsequent development of MDR-TB. Early detection of the patients with INH resistant strains would facilitate the modification of treatment regimens and appropriate infection control measures can be taken in time to reduce the risk of further development and transmission of MDR-TB.

## List of Abbreviations

MTB: *Mycobacterium tuberculosis*; MDR: multidrug-resistant; INH: isoniazid; RIF: rifampicin; STR: streptomycin; EMB: ethambutol; RFLP: restriction fragment length polymorphism; SIT: spoligotype international type.

## Competing interests

The authors declare that they have no competing interests.

## Authors' contributions

YH carried out the data collection and molecular genotyping studies, participated in the PCR and sequence alignment and drafted the manuscript. SH has revised it critically for important intellectual content. WJ was involved in all the microbiological research. WW participated in the conception and design, acquisition of data, its analysis and interpretation. BX conceived the study, developed the design, coordinated the implementation, and helped to revise the manuscript. All authors read and approved the final manuscript.

## Pre-publication history

The pre-publication history for this paper can be accessed here:

http://www.biomedcentral.com/1471-2334/10/43/prepub

## References

[B1] Espinal MA Laszlo A Simonsen L Boulahbal F Kim SJ Reniero A Hoffner S Rieder HL Binkin N Dye C Global trends in resistance to antituberculosis drugs. World Health Organization-International Union against Tuberculosis and Lung Disease Working Group on Anti-Tuberculosis Drug Resistance Surveillance N Engl J Med 2001 344 1294 1303 10.1056/NEJM200104263441706 11320389

[B2] Aziz MA Wright A Laszlo A De Muynck A Portaels F Van Deun A Wells C Nunn P Blanc L Raviglione M Epidemiology of antituberculosis drug resistance (the Global Project on Anti-tuberculosis Drug Resistance Surveillance): an updated analysis Lancet 2006 368 2142 2154 10.1016/S0140-6736(06)69863-2 17174706

[B3] WHO The WHO/IUATLD Global project on anti-tuberculosis drug resistance surveilance. Anti-tuberculosis drug resistance in the world. Report No.4 *WHO/HTM/TB/2008394* Geneva, Switzerland

[B4] Dye C Espinal MA Will tuberculosis become resistant to all antibiotics? Proc Biol Sci 2001 268 45 52 10.1098/rspb.2000.1328 12123297 PMC1087599

[B5] Zhang Y Heym B Allen B Young D Cole S The catalase-peroxidase gene and isoniazid resistance of Mycobacterium tuberculosis Nature 1992 358 591 593 10.1038/358591a0 1501713

[B6] Banerjee A Dubnau E Quemard A Balasubramanian V Um KS Wilson T Collins D de Lisle G Jacobs WR Jr inhA, a gene encoding a target for isoniazid and ethionamide in Mycobacterium tuberculosis Science 1994 263 227 230 10.1126/science.8284673 8284673

[B7] Heym B Alzari PM Honore N Cole ST Missense mutations in the catalase-peroxidase gene, katG, are associated with isoniazid resistance in Mycobacterium tuberculosis Mol Microbiol 1995 15 235 245 10.1111/j.1365-2958.1995.tb02238.x 7746145

[B8] Musser JM Kapur V Williams DL Kreiswirth BN van Soolingen D van Embden JD Characterization of the catalase-peroxidase gene (katG) and inhA locus in isoniazid-resistant and -susceptible strains of Mycobacterium tuberculosis by automated DNA sequencing: restricted array of mutations associated with drug resistance J Infect Dis 1996 173 196 202 8537659 10.1093/infdis/173.1.196

[B9] Zhang M Yue J Yang YP Zhang HM Lei JQ Jin RL Zhang XL Wang HH Detection of mutations associated with isoniazid resistance in Mycobacterium tuberculosis isolates from China J Clin Microbiol 2005 43 5477 5482 10.1128/JCM.43.11.5477-5482.2005 16272473 PMC1287806

[B10] van Soolingen D Qian L de Haas PE Douglas JT Traore H Portaels F Qing HZ Enkhsaikan D Nymadawa P van Embden JD Predominance of a single genotype of Mycobacterium tuberculosis in countries of east Asia J Clin Microbiol 1995 33 3234 3238 8586708 10.1128/jcm.33.12.3234-3238.1995 PMC228679

[B11] Hu Y Mathema B Wang W Hoffner S Kreiswirth B Xu B Prevalence of multidrug-resistant pulmonary tuberculosis in counties with different duration of DOTS implementation in rural China Microb Drug Resist 2008 14 227 232 10.1089/mdr.2008.0823 18707239

[B12] Canetti G Froman S Grosset J Hauduroy P Langerova M Mahler HT Meissner G Mitchison DA Sula L Mycobacteria: Laboratory Methods for Testing Drug Sensitivity and Resistance Bull World Health Organ 1963 29 565 578 14102034 PMC2555065

[B13] van Embden JD Cave MD Crawford JT Dale JW Eisenach KD Gicquel B Hermans P Martin C McAdam R Shinnick TM Strain identification of Mycobacterium tuberculosis by DNA fingerprinting: recommendations for a standardized methodology J Clin Microbiol 1993 31 2 406 409 8381814 10.1128/jcm.31.2.406-409.1993 PMC262774

[B14] Vitol I Driscoll J Kreiswirth B Kurepina N Bennett KP Identifying Mycobacterium tuberculosis complex strain families using spoligotypes Infect Genet Evol 2006 6 6 491 504 10.1016/j.meegid.2006.03.003 16632413

[B15] Dobner P Rusch-Gerdes S Bretzel G Feldmann K Rifai M Loscher T Rinder H Usefulness of Mycobacterium tuberculosis genomic mutations in the genes katG and inhA for the prediction of isoniazid resistance Int J Tuberc Lung Dis 1997 1 365 369 9432394

[B16] Escalante P Ramaswamy S Sanabria H Soini H Pan X Valiente-Castillo O Musser JM Genotypic characterization of drug-resistant Mycobacterium tuberculosis isolates from Peru Tuber Lung Dis 1998 79 111 118 10.1054/tuld.1998.0013 10645449

[B17] Marttila HJ Soini H Eerola E Vyshnevskaya E Vyshnevskiy BI Otten TF Vasilyef AV Viljanen MK A Ser315Thr substitution in KatG is predominant in genetically heterogeneous multidrug-resistant Mycobacterium tuberculosis isolates originating from the St. Petersburg area in Russia Antimicrob Agents Chemother 1998 42 2443 2445 9736581 10.1128/aac.42.9.2443 PMC105851

[B18] Lee AS Lim IH Tang LL Telenti A Wong SY Contribution of kasA analysis to detection of isoniazid-resistant Mycobacterium tuberculosis in Singapore Antimicrob Agents Chemother 1999 43 2087 2089 10428945 10.1128/aac.43.8.2087 PMC89423

[B19] Varela G Gonzalez S Gadea P Coitinho C Mota I Gonzalez G Goni F Rivas C Schelotto F Prevalence and dissemination of the Ser315Thr substitution within the KatG enzyme in isoniazid-resistant strains of Mycobacterium tuberculosis isolated in Uruguay J Med Microbiol 2008 57 1518 1522 10.1099/jmm.0.2008/001917-0 19018023

[B20] Fang Z Doig C Rayner A Kenna DT Watt B Forbes KJ Molecular evidence for heterogeneity of the multiple-drug-resistant Mycobacterium tuberculosis population in Scotland (1990 to 1997) J Clin Microbiol 1999 37 998 1003 10074516 10.1128/jcm.37.4.998-1003.1999 PMC88639

[B21] Chen X Ma Y Jin Q Jiang GL Li CY Wang Q Characterization of the katG, inhA, ahpC, kasA, and oxyR gene mutations in isoniazid-resistant and susceptible strain of Mycobacterium tuberculosis by automated DNA sequencing Zhonghua Jie He He Hu Xi Za Zhi 2005 28 250 253 15854436

[B22] Bakonyte D Baranauskaite A Cicenaite J Sosnovskaja A Stakenas P Molecular characterization of isoniazid-resistant Mycobacterium tuberculosis clinical isolates in Lithuania Antimicrob Agents Chemother 2003 47 2009 2011 10.1128/AAC.47.6.2009-2011.2003 12760887 PMC155844

[B23] Hillemann D Kubica T Agzamova R Venera B Rusch-Gerdes S Niemann S Rifampicin and isoniazid resistance mutations in Mycobacterium tuberculosis strains isolated from patients in Kazakhstan Int J Tuberc Lung Dis 2005 9 1161 1167 16229229

[B24] Piatek AS Telenti A Murray MR El-Hajj H Jacobs WR Jr Kramer FR Alland D Genotypic analysis of Mycobacterium tuberculosis in two distinct populations using molecular beacons: implications for rapid susceptibility testing Antimicrob Agents Chemother 2000 44 103 110 10.1128/AAC.44.1.103-110.2000 10602730 PMC89635

[B25] Huang H Jin Q Ma Y Chen X Zhuang Y Characterization of rpoB mutations in rifampicin-resistant Mycobacterium tuberculosis isolated in China Tuberculosis (Edinb) 2002 82 79 83 10.1054/tube.2002.0326 12356458

[B26] Ahmad S Fares E Genotypic diversity among isoniazid-resistant isolates of Mycobacterium tuberculosis from Rashid hospital in Dubai, United Arab Emirates Med Princ Pract 2005 14 16 21 10.1159/000081918 15608476

[B27] Caws M Duy PM Tho DQ Lan NT Hoa DV Farrar J Mutations prevalent among rifampin- and isoniazid-resistant Mycobacterium tuberculosis isolates from a hospital in Vietnam J Clin Microbiol 2006 44 2333 2337 10.1128/JCM.00330-06 16825345 PMC1489476

[B28] Mokaddas E Ahmad S Abal AT Molecular fingerprinting of isoniazid-resistant Mycobacterium tuberculosis isolates from chest diseases hospital in Kuwait Microbiol Immunol 2002 46 767 771 12516773 10.1111/j.1348-0421.2002.tb02762.x

[B29] Ano H Matsumoto T Suetake T Nagai T Tamura Y Takamatsu I Iwasaki T Matsuoka H Sasada S Tetsumoto S Relationship between the isoniazid-resistant mutation katGS315T and the prevalence of MDR-/XDR-TB in Osaka, Japan Int J Tuberc Lung Dis 2008 12 1300 1305 18926041

[B30] Kruuner A Hoffner SE Sillastu H Danilovits M Levina K Svenson SB Ghebremichael S Koivula T Kallenius G Spread of drug-resistant pulmonary tuberculosis in Estonia J Clin Microbiol 2001 39 3339 3345 10.1128/JCM.39.9.3339-3345.2001 11526173 PMC88341

